# Supplementary material for: Harnessing Attenuation-Related Mutations of Viral Genomes: Development of a Serological Assay to Differentiate between Capripoxvirus-Infected and -Vaccinated Animals
Source: Viruses. 2023 Nov 25;15(12):2318. doi: 10.3390/v15122318 (PMC10747038; doi:10.3390/v15122318)
Supplement: Supplementary file 1 [file viruses-15-02318-s001.zip › viruses-2701224-supplementary.pdf]

viruses MDPI

Supplementary Figures

Harnessing Attenuation-related Mutations of Viral Genomes: Development of a Serological Assay to Differentiate between Capripoxvirus Infected and Vaccinated Animals_ Francisco J. Berguido et al.

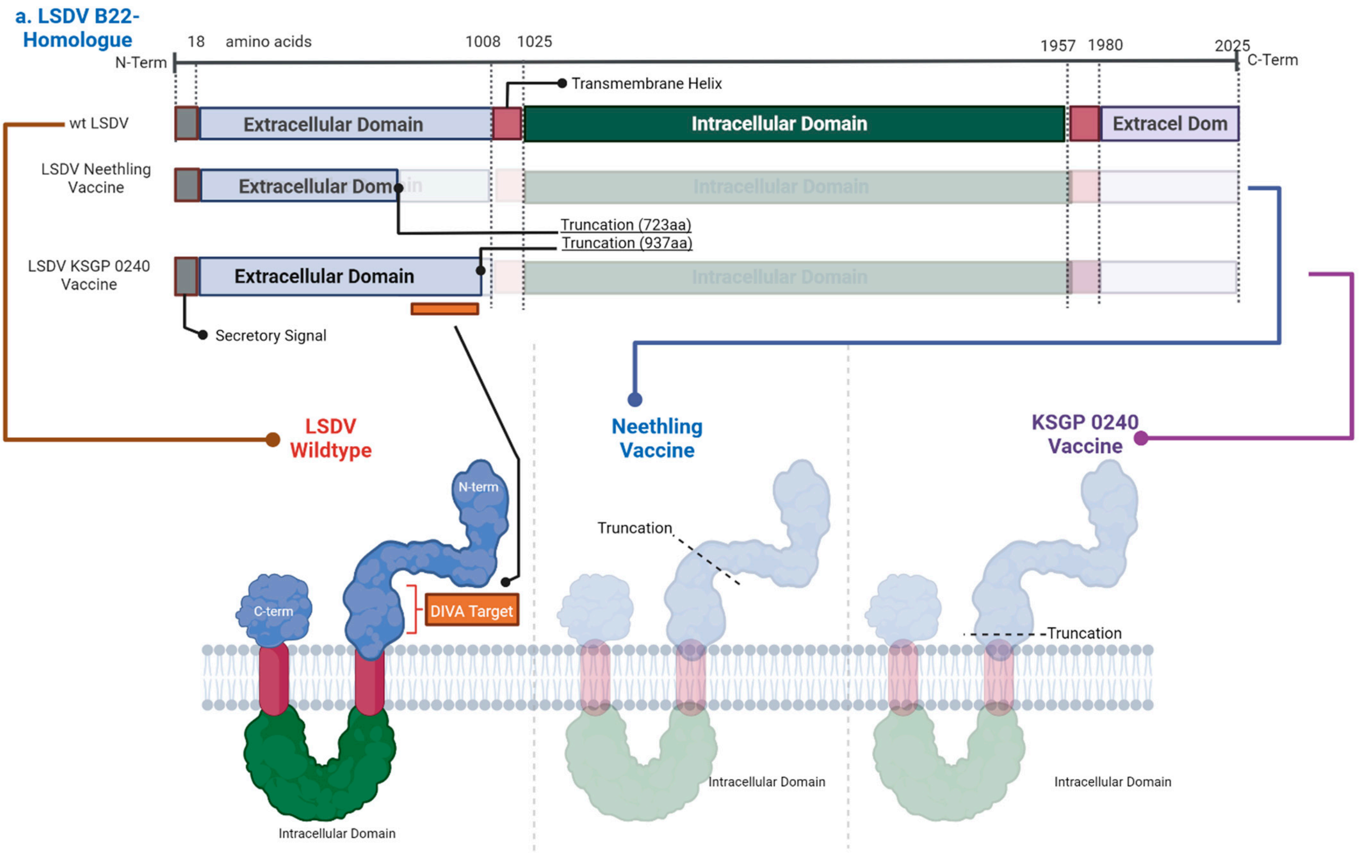

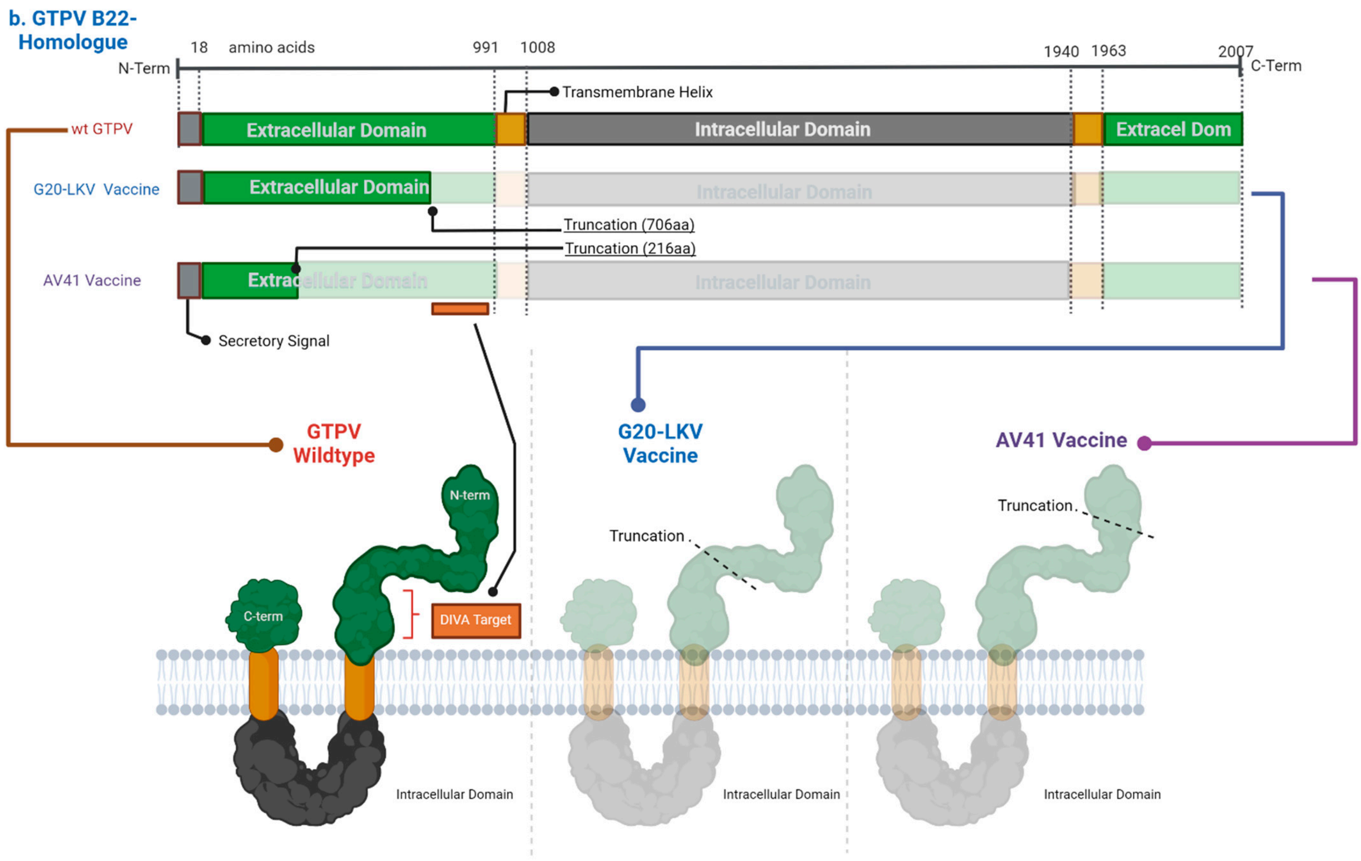

Figure S1. Domain predictions of wildtype and vaccine B22R gene products of (a) LSDV and (b) GTPV. Vaccine-related changes would result in a truncated or absent protein. Square bracket indicates the DIVA target region.

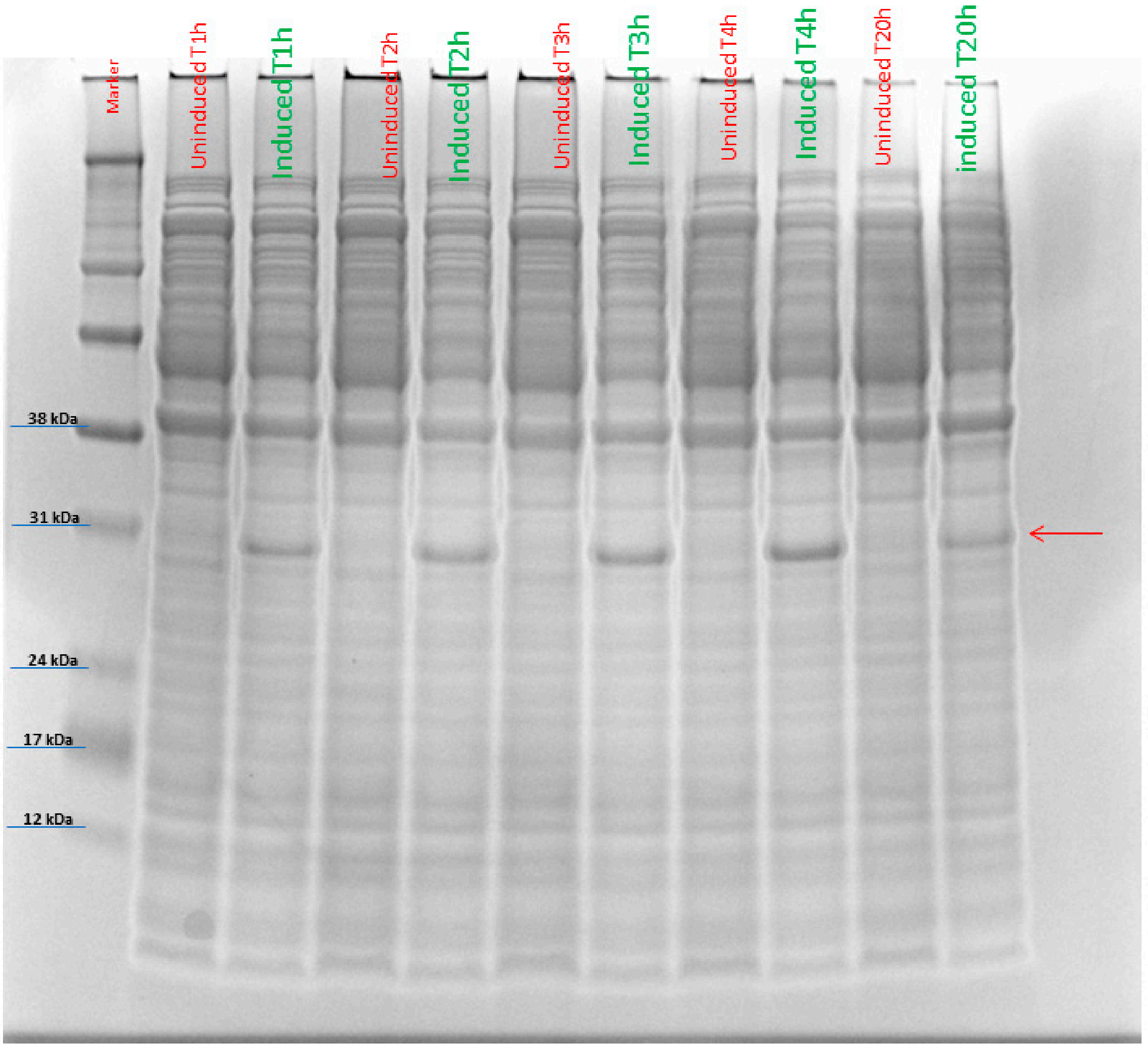

Figure S2. Time induction of WTS Diva Target Protein Fragment. The protein fragment expression was induced by arabinose and IPTG in BL21 cells. The observed fragment size was 28kDa (red arrow).

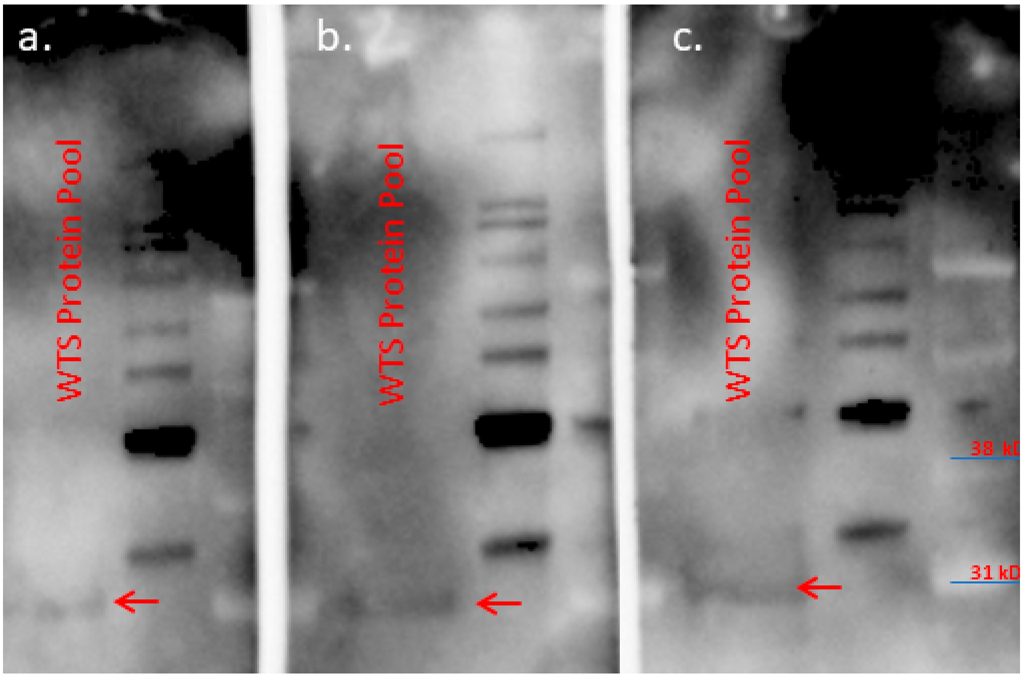

Figure S3. Western blot of Expressed WTS Diva Protein Fragment using anti-Sheeppox and anti-Goatpox positive sera. (a). Lane 1. Purified WTS Elution Pool, lane 2. Magic marker, lane 3. Rainbow marker probed with a. MALI Djelfa Sheep 1 terminal bleed positive serum, (b). Experimentally infected Goatpox positive serum (dpi. 21), (c). MALI Djelfa Sheep 6 positive serum (dpi 21). The observed fragment size is indicated by the red arrow.

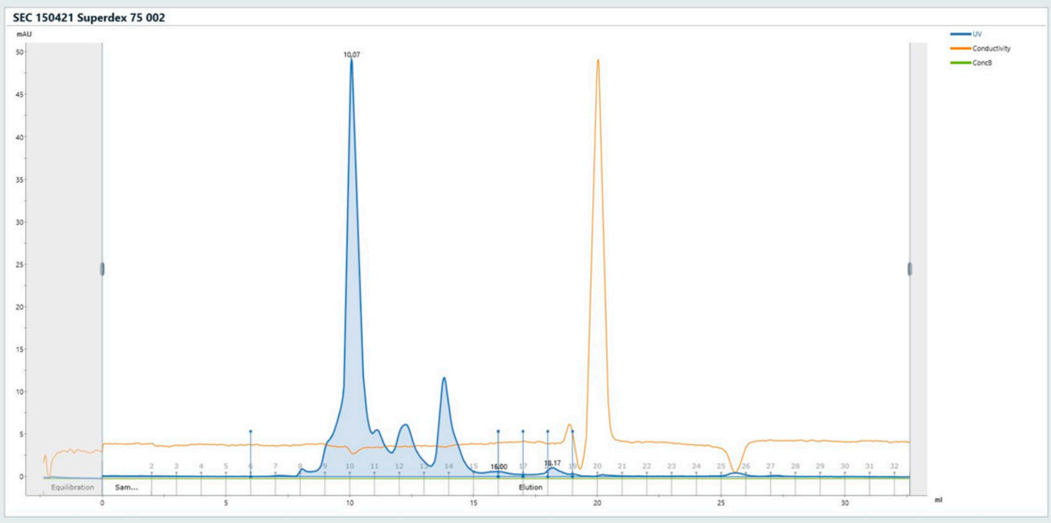

(a)

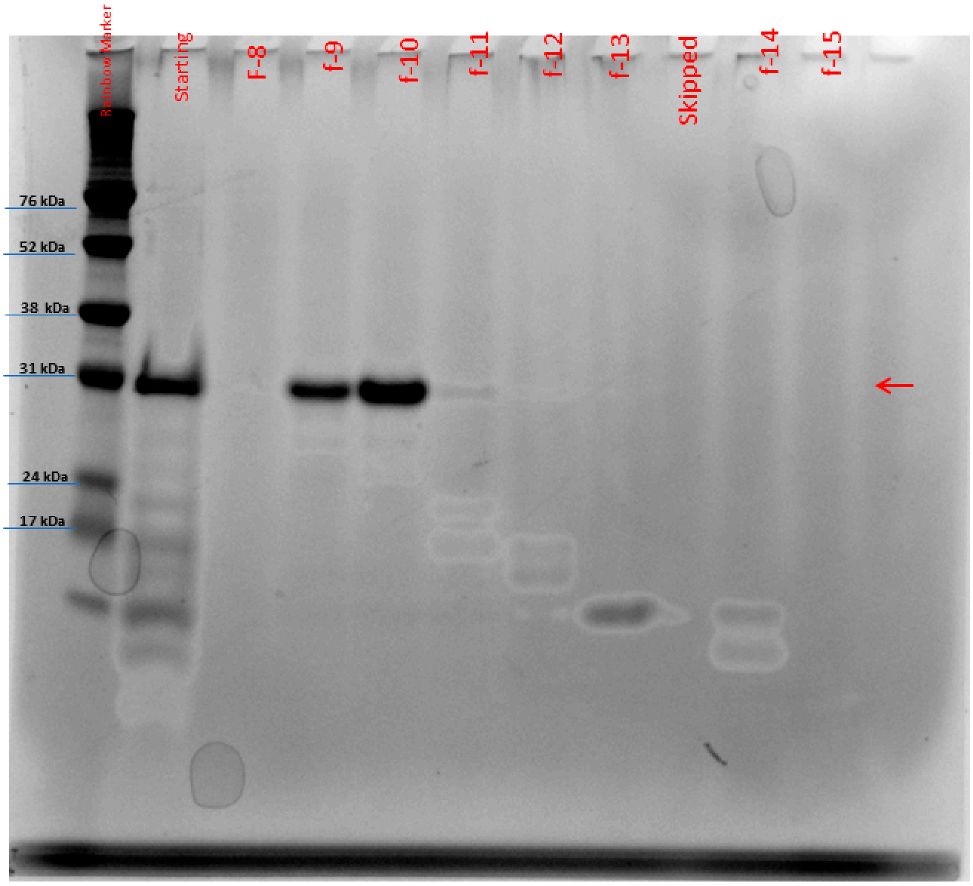

(b)

Figure S4. (a). ÄKTA Protein Purification Elution Graph. Typical WTS protein fragment purification graph using the Superdex increase 75 column. (b). SDS-PAGE of Purified WTS Protein Fragment. Elution fractions from ÄKTA. The collected elution peaks were fractions 9 and 10 (red arrow).

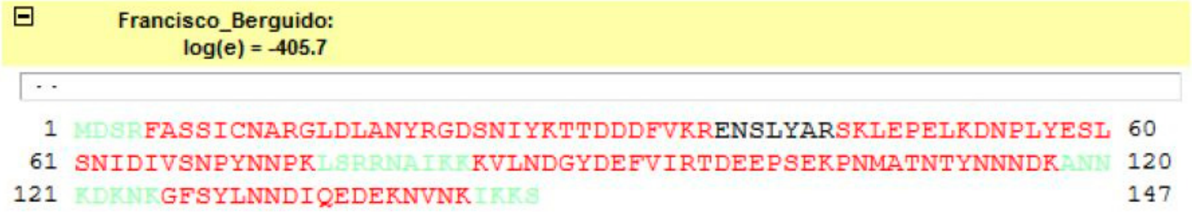

Figure S5. MS/MS Results. Sequences highlighted in red were identified by mass spect (MS/MS), sequences in green or black were not identified (green are unlikely to be found).

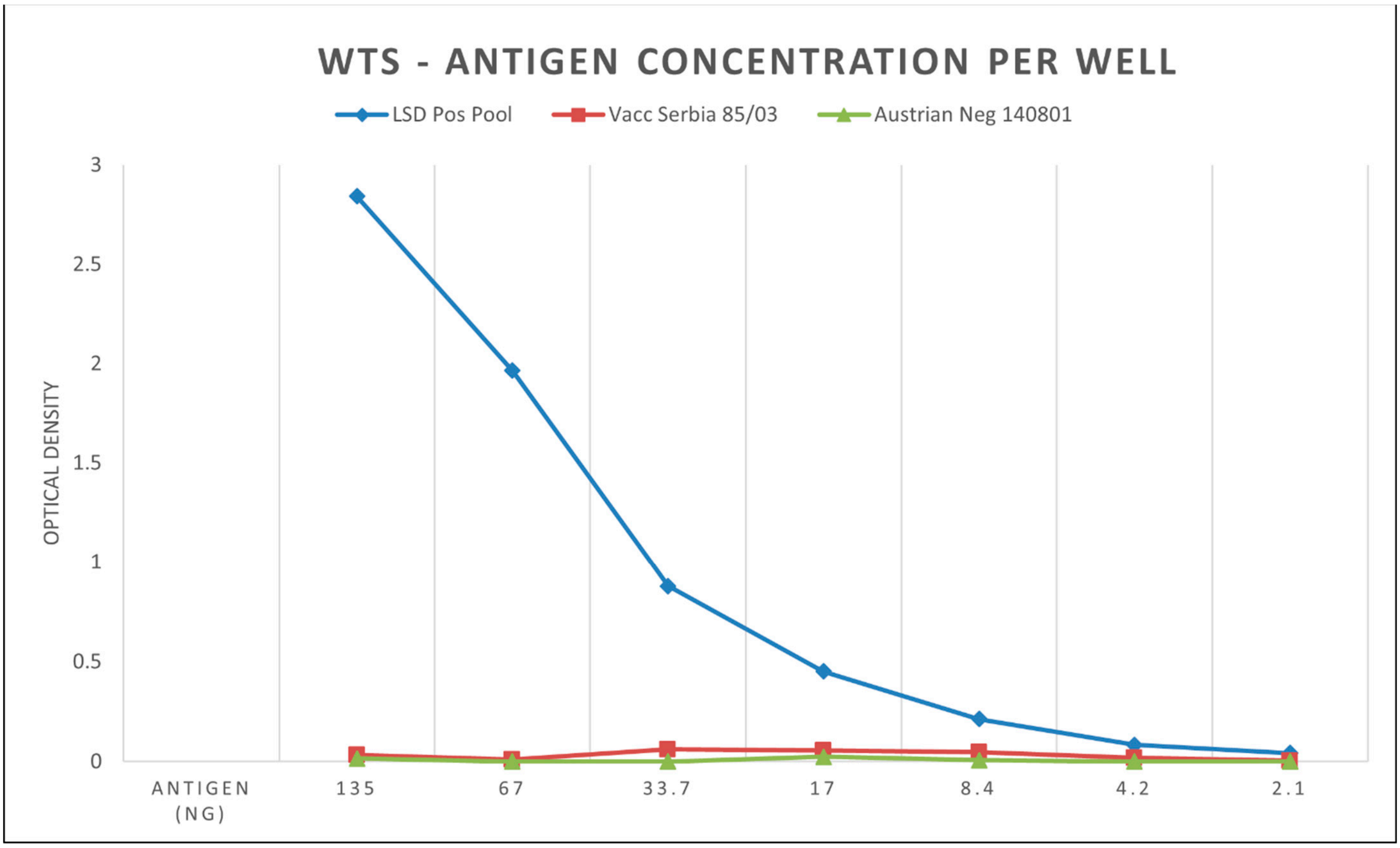

Figure S6. With constant serum (1 in 100) and secondary dilution (1 in 15000), wts protein fragment concentration (ng) per well was tested against positive serum (LSD pool) and negative sera (Serbia 85/03 and Austrian Neg). 60ng/well was selected.

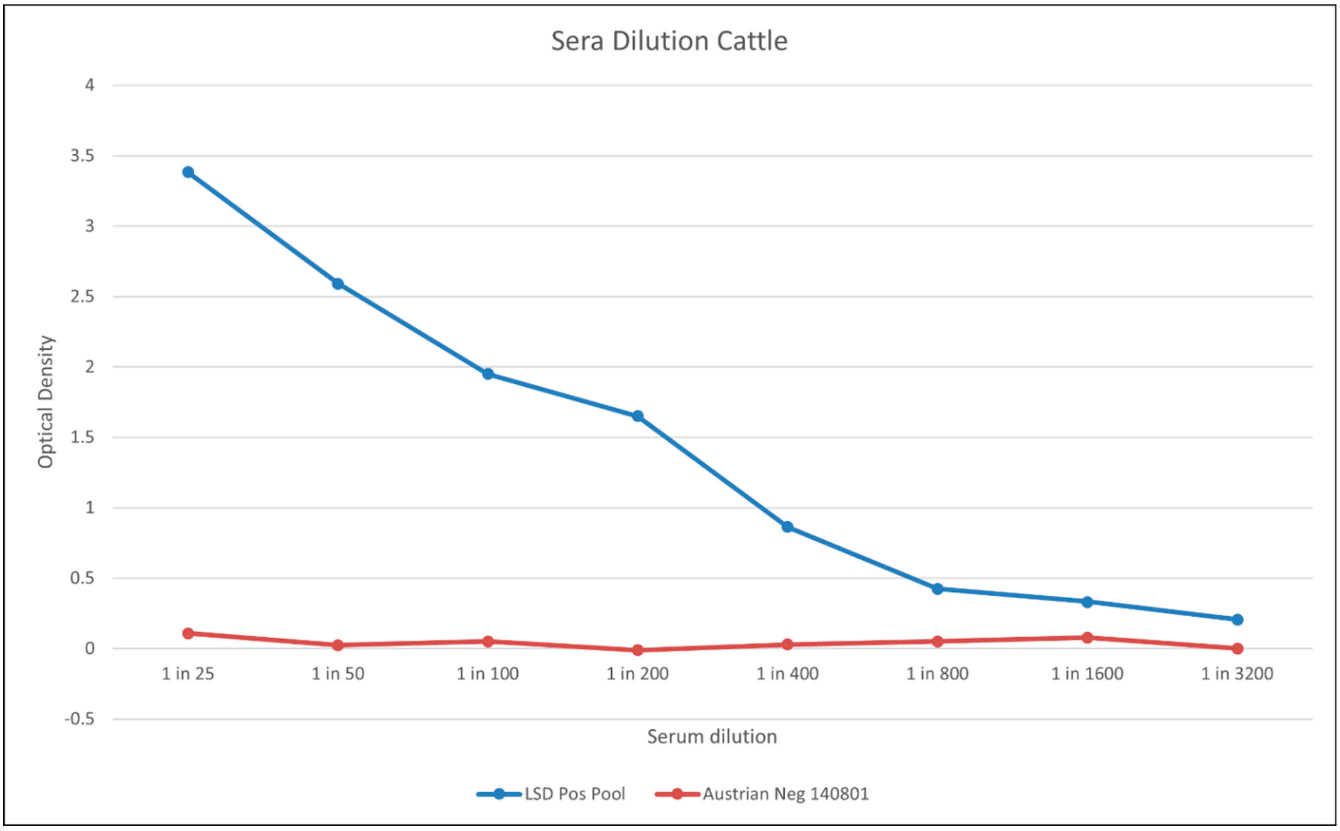

(a)

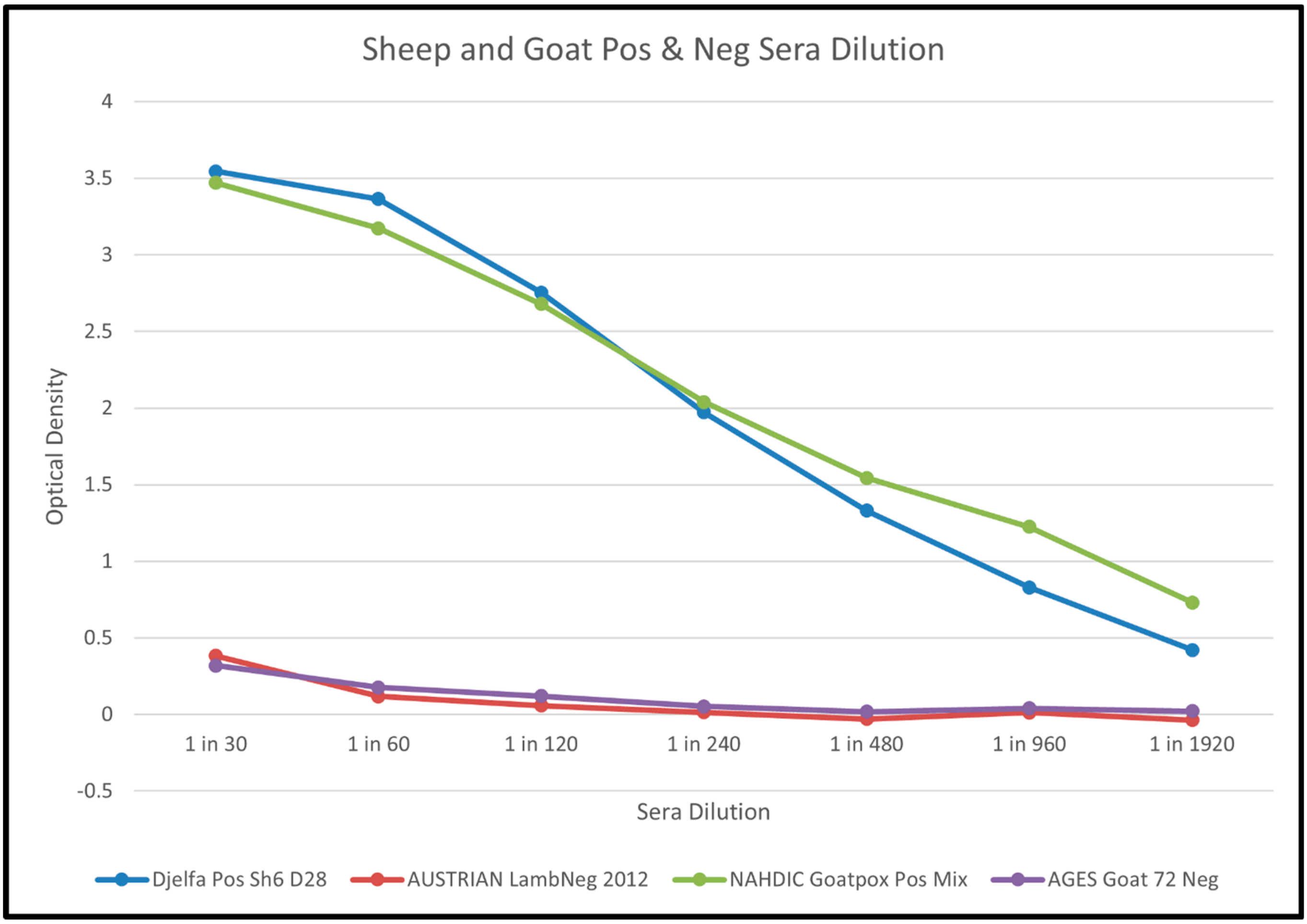

(b)

Figure S7. (a). Chessboard Titration of Positive and Negative LSD Sera. WTS protein fragment concentration was maintained constant (60ng/well). A serum dilution of 1 in 100 was selected for cattle. (b). Chessboard Titration of SPP and GTP Positive and Negative Sera. WTS protein fragment concentration was maintained constant (60ng/well). A serum dilution of 1 in 400 was selected for sheep and goat.

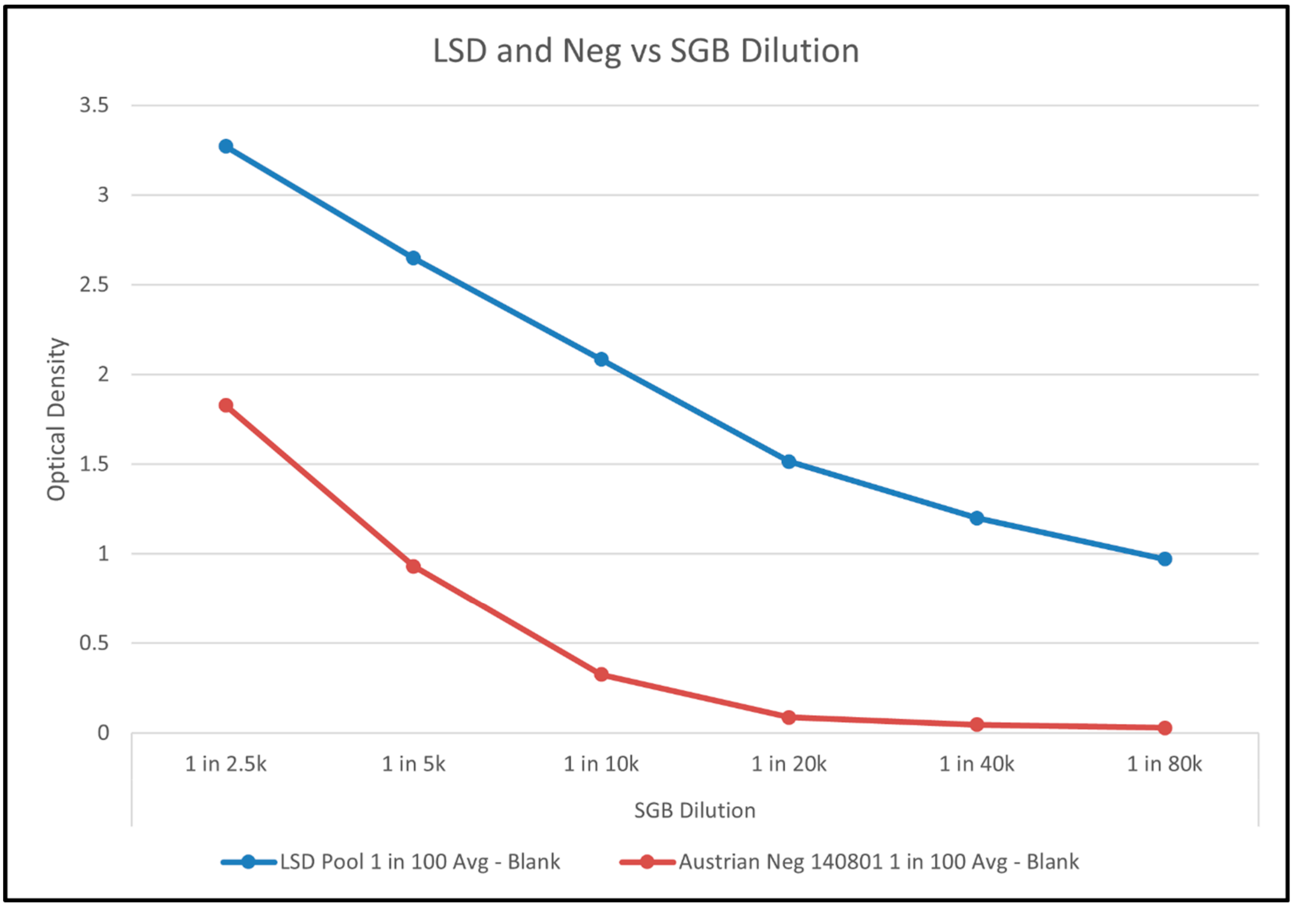
LSD and Neg vs SGB Dilution
Optical Density
3.5
3
2.5
2
1.5
1
0.5
0
1 in 2.5k
1 in 5k
1 in 10k
1 in 20k
1 in 40k
1 in 80k
SGB Dilution
LSD Pool 1 in 100 Avg - Blank
Austrian Neg 140801 1 in 100 Avg - Blank

(a)

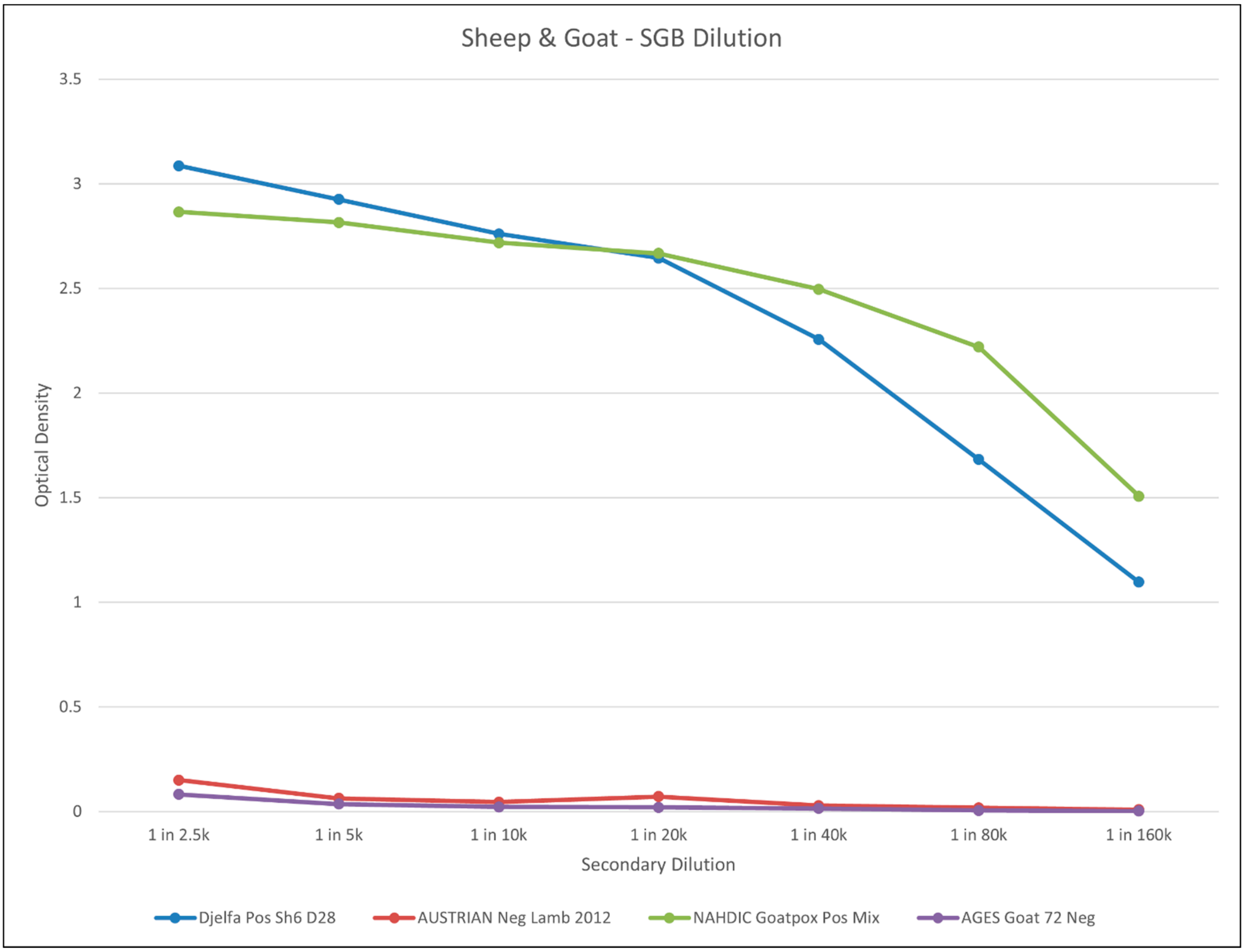

(b)

Figure S8. (a). Graph of Serial Dilution of Secondary (SGB) Antibody against LSD Positive and Negative Sera. Antigen (60ng/well) and Sera (1 in 100) were kept constant. A secondary dilution of 1 in 30000 was selected for cattle. (b). Graph of Serial Dilution of Secondary (SGB) Antibody against SPP and GTP Positive and Negative Sera. Antigen (60ng/well) and Sera (1 in 400) were kept constant. A secondary dilution of 1 in 20000 was selected for cattle.

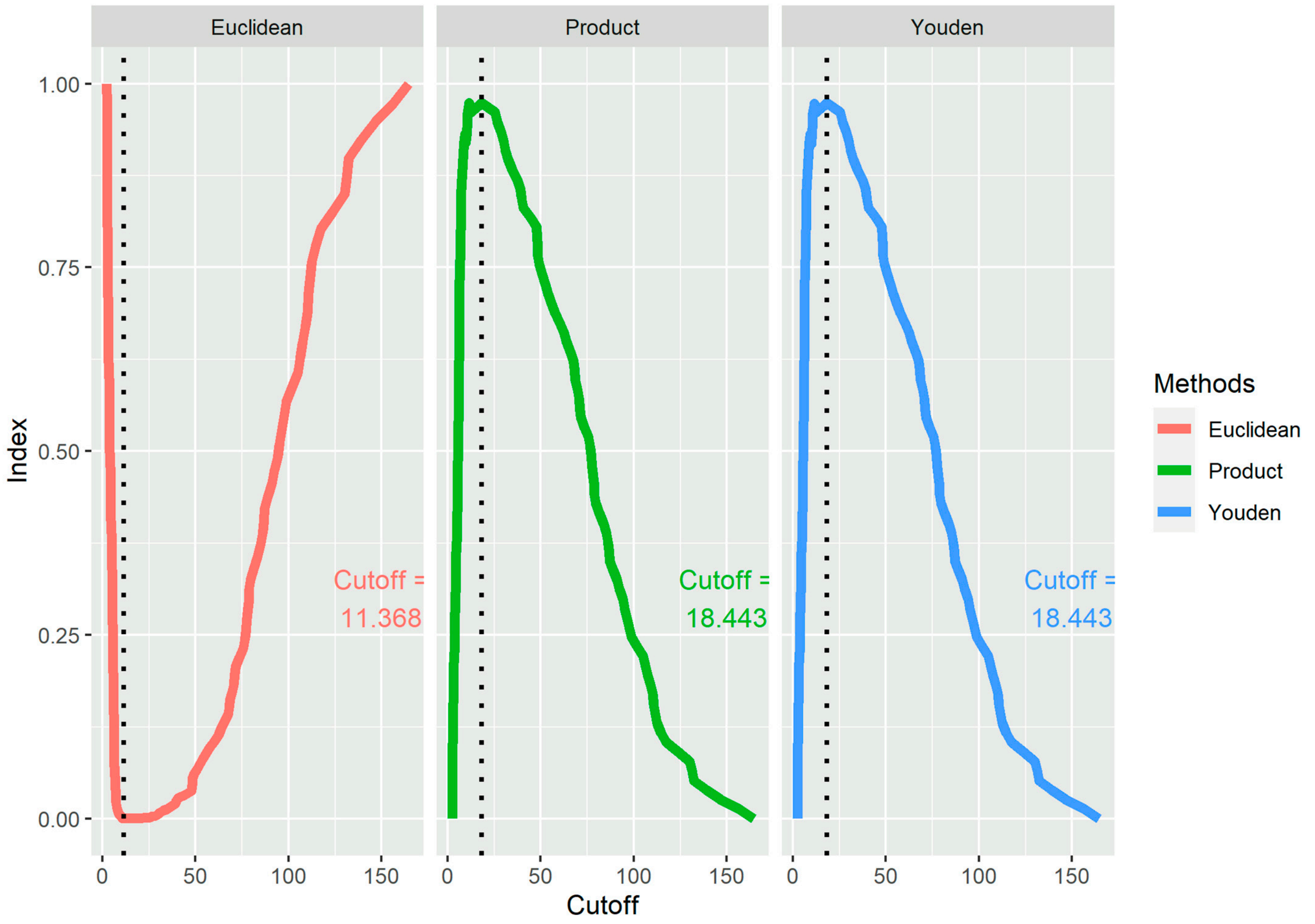

Figure S9. Establishment of cut-off for LSDV determined by ROC analysis and Euclidean, Product and Youden indexes. We chose the value of Youden and Product indexes, over the Euclidean's, as they both agreed (18.443).

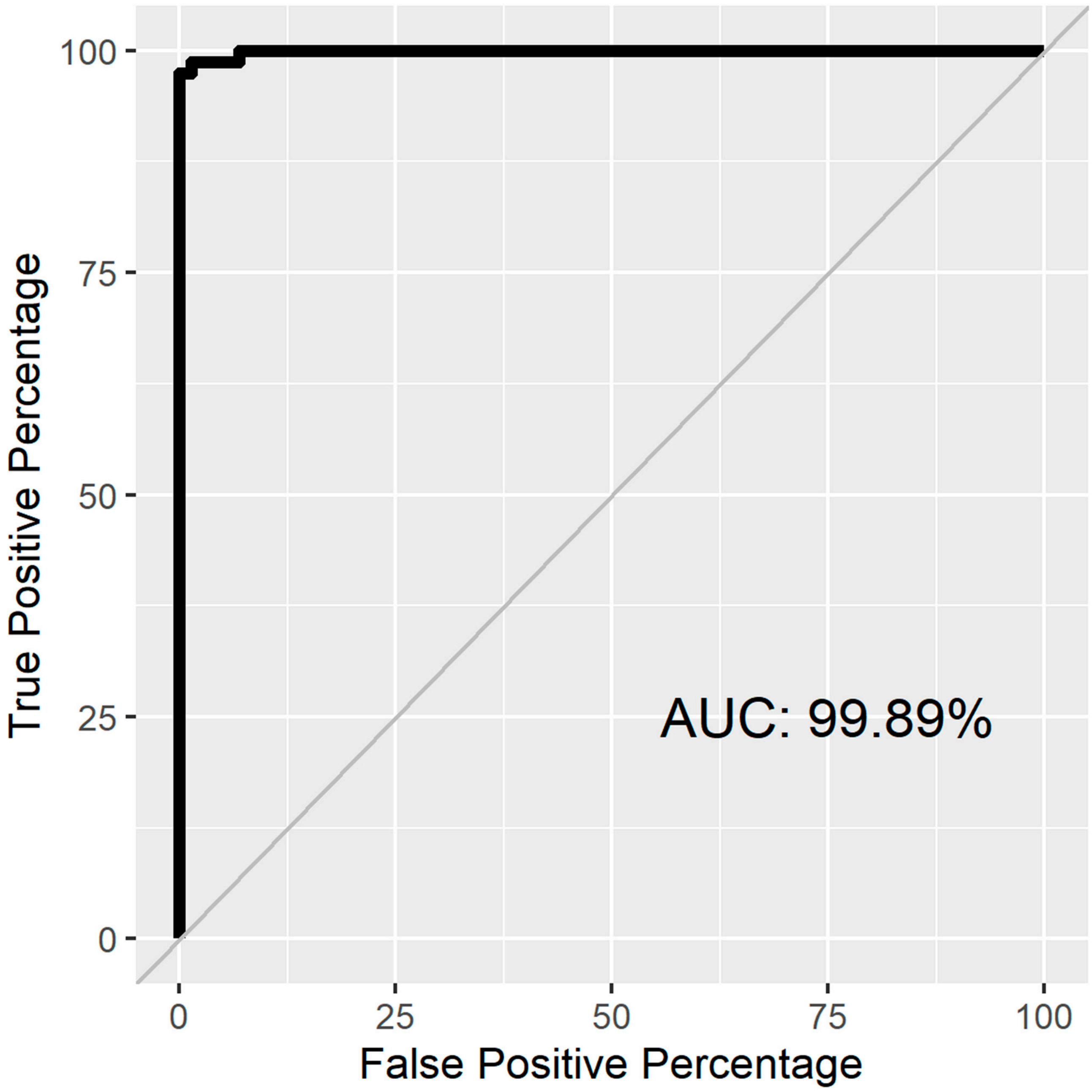

Figure S10. ROC analysis of the LSD wts-DIVA iELISA. A calculation of 99.89% of the area under the curve was established for the test.

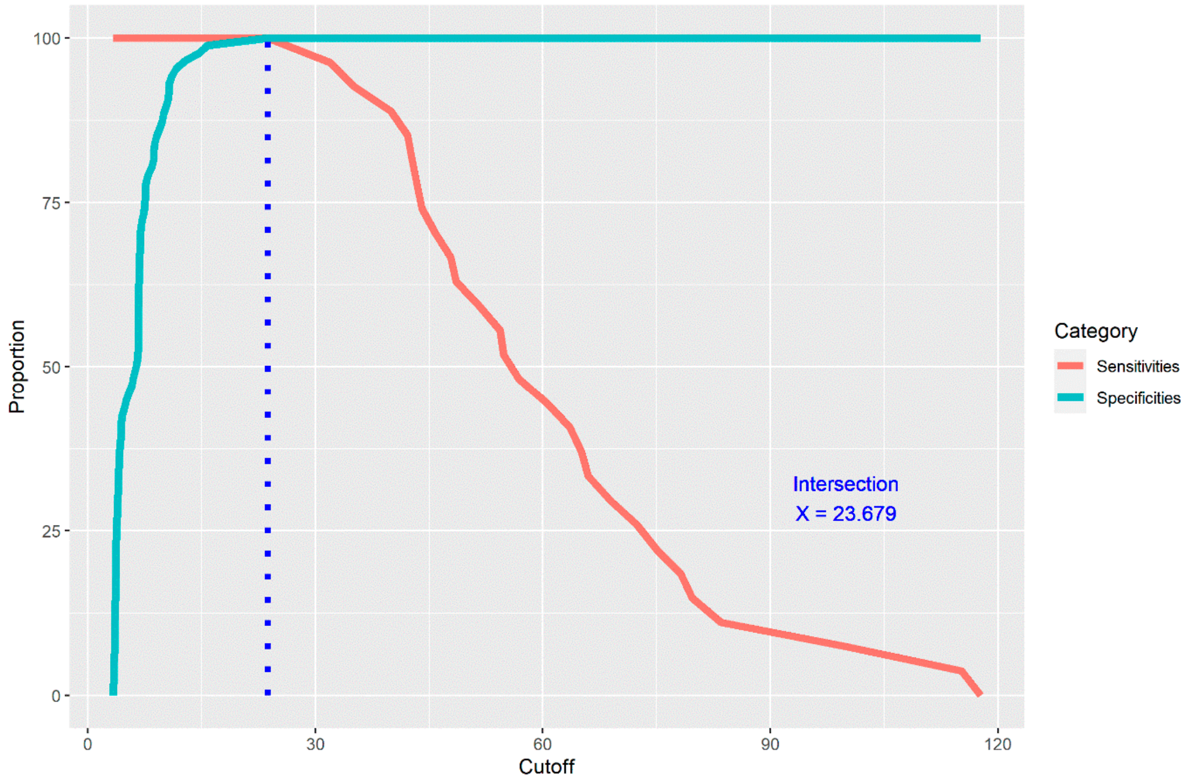

Figure S11. Calculation of cut-off for SPP and GTP (23.679) determined by ROC analysis and Euclidean, Product and Youden indexes.

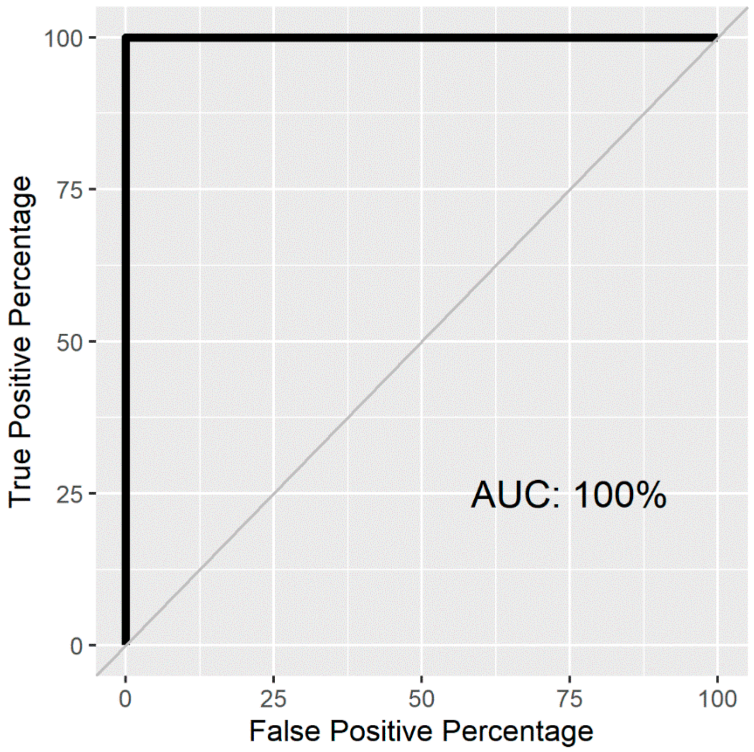

Figure S12. ROC analysis of the SPP/GTP wts-DIVA iELISA. A calculation of 99.89% of the area under the curve was established for the test.

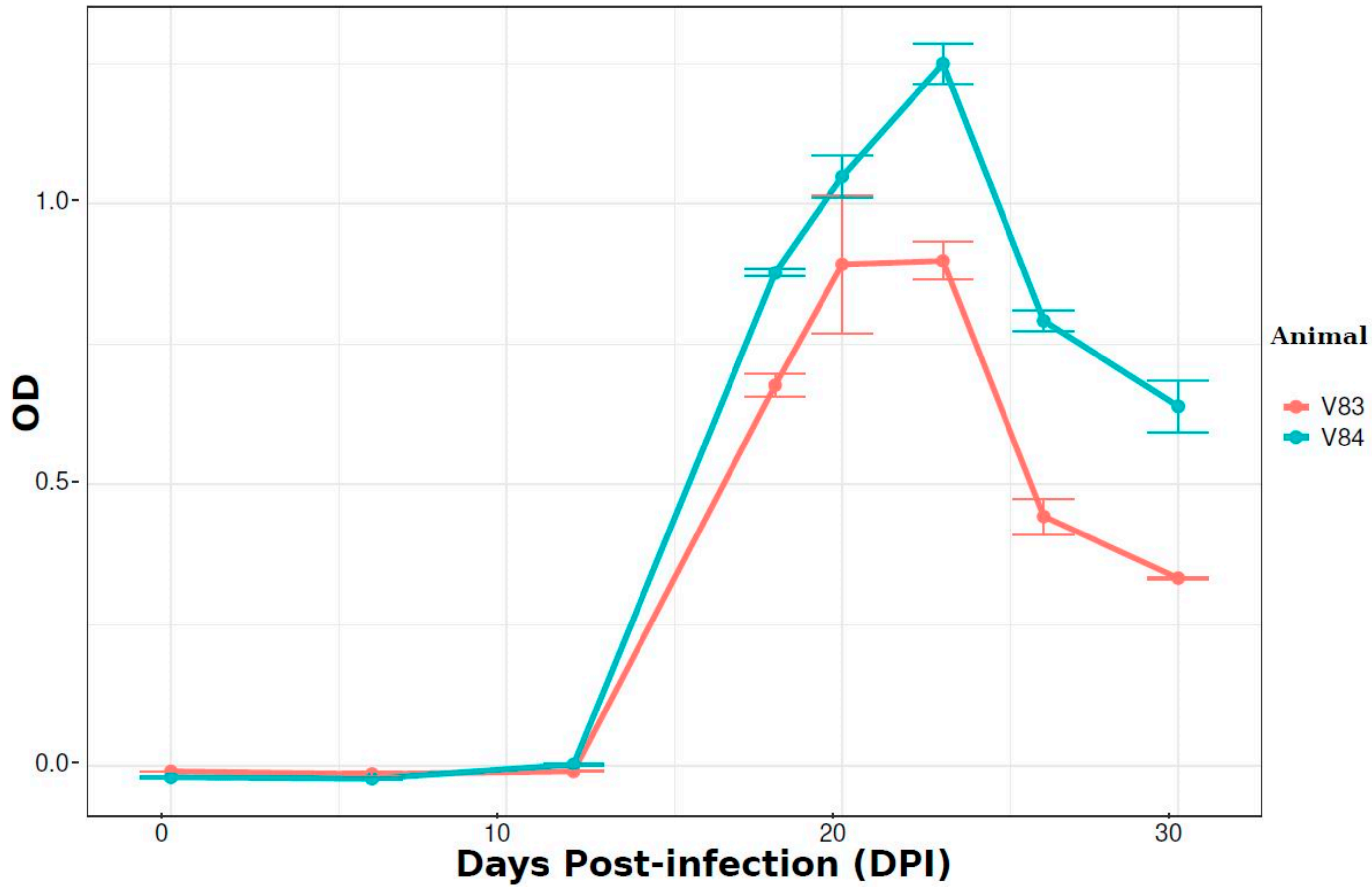

Figure S13. Samples from cattle infected with virulent South African LSDV Neethling strain. The seroconversion occurred between 8 DPI and 12 DPI with the highest detection level at 23 DPI. Antibodies were detected at every point after 12 DPI, including the last collected data point at 30 DPI.

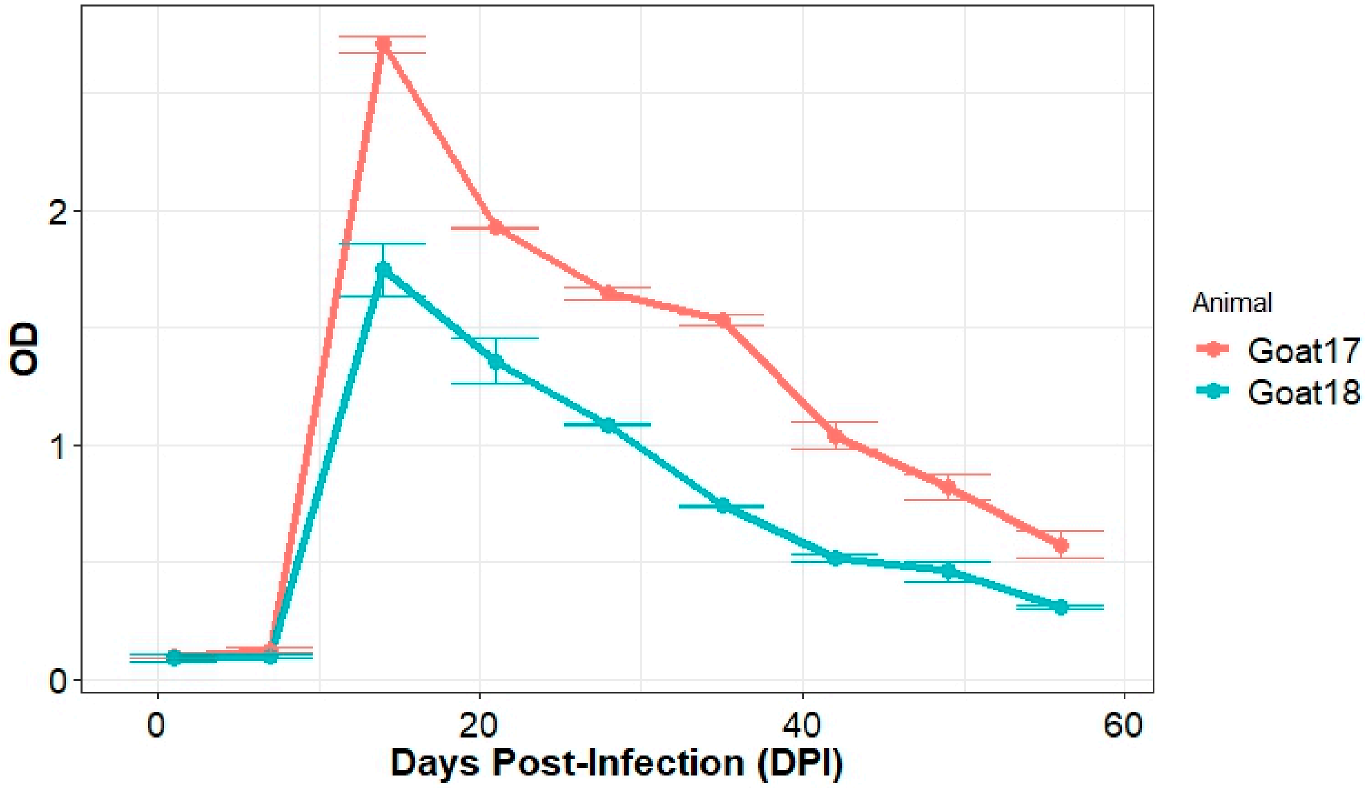

Figure S14. Samples from goats infected using GTPV Oman 84, seroconversion occurred between 7 DPI and 14 DPI with the highest detection level at 14 DPI. The goats remained positive from 14 DPI to at least 49 DPI.

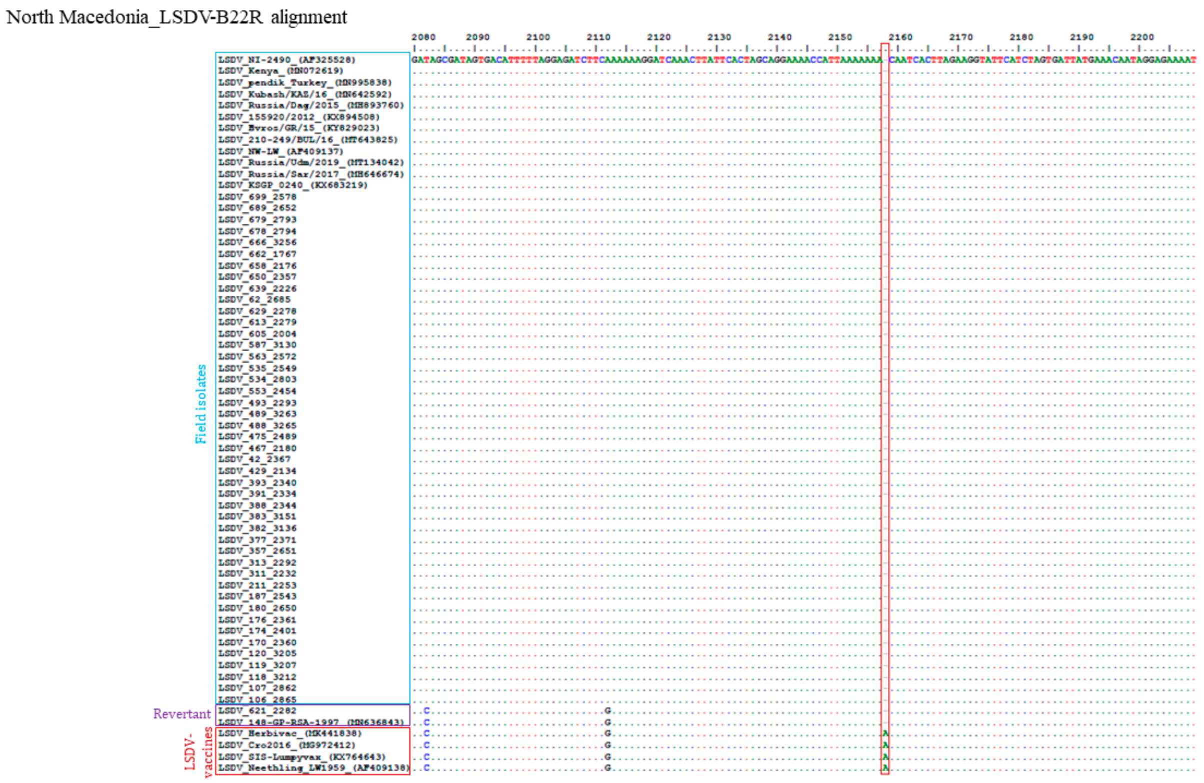

Figure S15. Sequence alignment of a fragment of the B22R gene of several North Macedonia outbreak samples. Sample 621, shows a vaccine-like profile but lacks the A insertion, typically found in the B22R gene of vaccine viruses derived from the Neethling vaccine strain. This suggests a sample reversion to virulence. Field LSDV 148 from South Africa, found online, showed a similar profile.

Table S1. Detail information on seventy-five vaccinated cattle samples that were confirmed positive by SNT and/or IDVet double antigen ELISA for capripox. Red fond indicates positive samples.

SERUM_ID		S/P% (wts_DIVA_ELISA)	wts_DIVA_ELISA_Result	VNT Dilution	VNT Result	Other Tests	IDVet ELISA	IDVet ELISA Results	Sample info
Serbian 4	Vaccinated (One Year Post Vaccination) - IDVet and or VNT Pos	3.1535	NEG	1in12	Pos	NA	60.28	IDVet Pos	Vaccinated (OBP) sample a year later
Serbian 6		2.8487	NEG	1in12	Pos	NA	NA	NA	Vaccinated (OBP) sample a year later
Serbian 10		2.6460	NEG	1in64	Pos	NA	206.78	IDVet Pos	Vaccinated (OBP) sample a year later
Serbian 13		3.0219	NEG	1in45	Pos	NA	134	IDVet Pos	Vaccinated (OBP) sample a year later
Serbian 14		2.8255	NEG	1in45	Pos	NA	186	IDVet Pos	Vaccinated (OBP) sample a year later
Serbian 20		2.7502	NEG	1in64	Pos	NA	144	IDVet Pos	Vaccinated (OBP) sample a year later
Serbian 24		2.7116	NEG	1in12	Pos	NA	NA	NA	Vaccinated (OBP) sample a year later
Serbian 27		2.8881	NEG	1in12	Pos	NA	53.8	IDVet Pos	Vaccinated (OBP) sample a year later
Serbian 35		6.1606	NEG	1in45	Pos	NA	83.1	IDVet Pos	Vaccinated (OBP) sample a year later
Serbian 36		2.8561	NEG	1in24	Pos	NA	156.1	IDVet Pos	Vaccinated (OBP) sample a year later
Serbian 38		2.6004	NEG	1in12	Pos	NA	62.3	IDVet Pos	Vaccinated (OBP) sample a year later
Serbian 39		2.7776	NEG	1in12	Pos	NA	62.1	IDVet Pos	Vaccinated (OBP) sample a year later
Serbian 42		2.9064	NEG	1in64	Pos	NA	21.5	IDVet Pos	Vaccinated (OBP) sample a year later
Serbian 43		3.0855	NEG	1in45	Pos	NA	77.6	IDVet Pos	Vaccinated (OBP) sample a year later
Serbian 44		3.1957	NEG	1in45	Pos	NA	87.5	IDVet Pos	Vaccinated (OBP) sample a year later
Serbian 53		3.2149	NEG	1in45	Pos	NA	86.9	IDVet Pos	Vaccinated (OBP) sample a year later
Serbian 54		2.7650	NEG	1in24	Pos	NA	19	IDVet Neg	Vaccinated (OBP) sample a year later
Serbian 61		2.5275	NEG	1in12	Pos	NA	NA	NA	Vaccinated (OBP) sample a year later
Serbian 62		2.6660	NEG	1in24	Pos	NA	69.8	IDVet Pos	Vaccinated (OBP) sample a year later
Serbian 65		2.8693	NEG	1in24	Pos	NA	NA	NA	Vaccinated (OBP) sample a year later
Serbian 66		2.6666	NEG	1in24	Pos	NA	107	IDVet Pos	Vaccinated (OBP) sample a year later
Serbian 67		2.7085	NEG	1in12	Pos	NA	NA	NA	Vaccinated (OBP) sample a year later
Serbian 73		3.0679	NEG	1in12	Pos	NA	58	IDVet Pos	Vaccinated (OBP) sample a year later
Serbian 75		2.6501	NEG	1in12	Pos	NA	64	IDVet Pos	Vaccinated (OBP) sample a year later
Serbian 78		2.3988	NEG	1in24	Pos	NA	103	IDVet Pos	Vaccinated (OBP) sample a year later
Serbian 79		2.6174	NEG	1in12	Pos	NA	72.9	IDVet Pos	Vaccinated (OBP) sample a year later
Serbian 81		2.8521	NEG	1in24	Pos	NA	60	IDVet Pos	Vaccinated (OBP) sample a year later
Serbian 88		2.5947	NEG	1in24	Pos	NA	52	IDVet Pos	Vaccinated (OBP) sample a year later
Serbian 90		2.8874	NEG	1in24	Pos	NA	133.7	IDVet Pos	Vaccinated (OBP) sample a year later
Serbian 91		2.6425	NEG	1in24	Pos	NA	82.6	IDVet Pos	Vaccinated (OBP) sample a year later
Serbian 93		2.7422	NEG	1in45	Pos	NA	85.8	IDVet Pos	Vaccinated (OBP) sample a year later
Serbian 94		2.6637	NEG	1in24	Pos	NA	42	IDVet Pos	Vaccinated (OBP) sample a year later
Serbian 95		3.2336	NEG	1in8	Pos	NA	30	IDVet Pos	Vaccinated (OBP) sample a year later
Serbian 98		6.8997	NEG	>1in64	Pos	NA	190	IDVet Pos	Vaccinated (OBP) sample a year later
Kraljevo 005	Vaccinated (8 Wks post Boost) - IDVet and or VNT Pos	5.7897	NEG	< 1in10	?	NA	45.37	IDVet Pos	vaccinated and a year later revaccinated and then sampled 8 weeks after that.
Kraljevo 006		6.8985	NEG	< 1in10	?	NA	272.00	IDVet Pos	vaccinated and a year later revaccinated and then sampled 8 weeks after that.
Kraljevo 007		5.1920	NEG	< 1in10	?	NA	51.96	IDVet Pos	vaccinated and a year later revaccinated and then sampled 8 weeks after that.
Kraljevo 008		2.9028	NEG	< 1in10	?	NA	46.33	IDVet Pos	vaccinated and a year later revaccinated and then sampled 8 weeks after that.
Kraljevo 012		5.0824	NEG	1in15	Pos	NA	201.85	IDVet Pos	vaccinated and a year later revaccinated and then sampled 8 weeks after that.
Kraljevo 013		4.6803	NEG	< 1in10	?	NA	34.80	IDVet Pos	vaccinated and a year later revaccinated and then sampled 8 weeks after that.
Kraljevo 017		3.5612	NEG	1in30	Pos	NA	295.88	IDVet Pos	vaccinated and a year later revaccinated and then sampled 8 weeks after that.
Kraljevo 018		2.6842	NEG	1in15	Pos	NA	188.95	IDVet Pos	vaccinated and a year later revaccinated and then sampled 8 weeks after that.
Kraljevo 020		2.6762	NEG	< 1in10	?	NA	75.70	IDVet Pos	vaccinated and a year later revaccinated and then sampled 8 weeks after that.
Kraljevo 021		3.7543	NEG	1in30	Pos	NA	169.60	IDVet Pos	vaccinated and a year later revaccinated and then sampled 8 weeks after that.
Kraljevo 022		6.4979	NEG	1in40	Pos	NA	117.71	IDVet Pos	vaccinated and a year later revaccinated and then sampled 8 weeks after that.
Kraljevo 028		4.1280	NEG	< 1in10	?	NA	56.90	IDVet Pos	vaccinated and a year later revaccinated and then sampled 8 weeks after that.
Kraljevo 029		7.2389	NEG	1in40	Pos	NA	44.27	IDVet Pos	vaccinated and a year later revaccinated and then sampled 8 weeks after that.
Kraljevo 030		3.6528	NEG	1in30	Pos	NA	141.46	IDVet Pos	vaccinated and a year later revaccinated and then sampled 8 weeks after that.
Kraljevo 031		4.0797	NEG	< 1in10	?	NA	31.50	IDVet Pos	vaccinated and a year later revaccinated and then sampled 8 weeks after that.
Kraljevo 033		3.5569	NEG	< 1in10	?	NA	80.23	IDVet Pos	vaccinated and a year later revaccinated and then sampled 8 weeks after that.
Kraljevo 037		3.8993	NEG	1in80	Pos	NA	259.09	IDVet Pos	vaccinated and a year later revaccinated and then sampled 8 weeks after that.
Kraljevo 038		3.2048	NEG	< 1in10	?	NA	49.49	IDVet Pos	vaccinated and a year later revaccinated and then sampled 8 weeks after that.
Kraljevo 039		3.0482	NEG	< 1in10	?	NA	197.87	IDVet Pos	vaccinated and a year later revaccinated and then sampled 8 weeks after that.
Kraljevo 040		4.4599	NEG	< 1in10	?	NA	80.78	IDVet Pos	vaccinated and a year later revaccinated and then sampled 8 weeks after that.
Kraljevo 042		4.7463	NEG	1in30	Pos	NA	91.21	IDVet Pos	vaccinated and a year later revaccinated and then sampled 8 weeks after that.
Kraljevo 047		15.8285	NEG	1in30	Pos	NA	207.48	IDVet Pos	vaccinated and a year later revaccinated and then sampled 8 weeks after that.
Kraljevo 050		8.5113	NEG	< 1in10	?	NA	103.43	IDVet Pos	vaccinated and a year later revaccinated and then sampled 8 weeks after that.
Kraljevo 051		3.6888	NEG	< 1in10	?	NA	33.15	IDVet Pos	vaccinated and a year later revaccinated and then sampled 8 weeks after that.
Kraljevo 054		4.0958	NEG	< 1in10	?	NA	88.61	IDVet Pos	vaccinated and a year later revaccinated and then sampled 8 weeks after that.
Kraljevo 057		9.4187	NEG	< 1in10	?	NA	122.79	IDVet Pos	vaccinated and a year later revaccinated and then sampled 8 weeks after that.
Kraljevo 064		3.1001	NEG	< 1in10	?	NA	144.06	IDVet Pos	vaccinated and a year later revaccinated and then sampled 8 weeks after that.
Kraljevo 067		4.0215	NEG	< 1in10	?	NA	98.63	IDVet Pos	vaccinated and a year later revaccinated and then sampled 8 weeks after that.
Kraljevo 069		2.7460	NEG	< 1in10	?	NA	178.79	IDVet Pos	vaccinated and a year later revaccinated and then sampled 8 weeks after that.
Kraljevo 074		2.8680	NEG	1in10	Pos	NA	98.49	IDVet Pos	vaccinated and a year later revaccinated and then sampled 8 weeks after that.
Kraljevo 077		3.2473	NEG	1in160	Pos	NA	247.56	IDVet Pos	vaccinated and a year later revaccinated and then sampled 8 weeks after that.
Kraljevo 079		4.7233	NEG	1in20	Pos	NA	143.65	IDVet Pos	vaccinated and a year later revaccinated and then sampled 8 weeks after that.
Kraljevo 083		5.6801	NEG	1in10	Pos	NA	195.54	IDVet Pos	vaccinated and a year later revaccinated and then sampled 8 weeks after that.
Kraljevo 086		3.4063	NEG	< 1in10	?	NA	41.94	IDVet Pos	vaccinated and a year later revaccinated and then sampled 8 weeks after that.
Kraljevo 088		5.1302	NEG	< 1in10	?	NA	54.98	IDVet Pos	vaccinated and a year later revaccinated and then sampled 8 weeks after that.
Kraljevo 090		4.1093	NEG	< 1in10	?	NA	169.18	IDVet Pos	vaccinated and a year later revaccinated and then sampled 8 weeks after that.
Kraljevo 093		3.8019	NEG	1in30	Pos	NA	42.93	IDVet Pos	vaccinated and a year later revaccinated and then sampled 8 weeks after that.
Kraljevo 095		7.0329	NEG	< 1in10	?	NA	140.46	IDVet Pos	vaccinated and a year later revaccinated and then sampled 8 weeks after that.
Kraljevo 096		2.9122	NEG	1in10-1in20	Pos	NA	27.08	IDVet Neg	vaccinated and a year later revaccinated and then sampled 8 weeks after that.
Kraljevo 099		3.8787	NEG	1in20	Pos	NA	173.68	IDVet Pos	vaccinated and a year later revaccinated and then sampled 8 weeks after that.
Kraljevo 100		4.7769	NEG	< 1in10	?	NA	36.84	IDVet Pos	vaccinated and a year later revaccinated and then sampled 8 weeks after that.

Table S2. 2016 North Macedonia LSD Outbreak Sample Details. The samples were tested by PCR, DIVA qPCR and the LSD wts-DIVA iELISA. Samples were sequenced by the Sanger method. Two animals (blue highlight) tested positive for field infection by DIVA qPCR and Sanger, positive by SNT, while tested negative for the wts-DIVA ELISA. Two animals (green highlight) tested positive for SNT and the wts-DIVA ELISA, and negative by DIVA qPCR and Sanger. One animal (orange highlight) tested positive for SNT and the wts-DIVA ELISA, but negative by DIVA qPCR and Sanger. One animal (grey highlight) tested positive for SNT and the wts-DIVA ELISA, mixed by DIVA qPCR and field infected by Sanger.

SERUM_ID	wts-DIVA ELISA	VNT Result	DIVA qPCR	Cq	Vaccinated? (yes/no)	Vaccine used	SampledPost Vaccination	Sanger Confirmed
LSDV_MKD_488	Positive	Positive	Field	Cq = 21.2	YES	Lumpyvax	31	Field
LSDV_MKD_489	Negative	Positive	Field	Cq = 16.9	YES	Lumpyvax	17	Field
LSDV_MKD_62	Positive	Positive	Field	Cq = 30.7	YES	OBP LSD vaccine	24	Field
LSDV_MKD_180	Positive	Positive	Field	Cq = 28.3	YES	OBP LSD vaccine	22	Field
LSDV_MKD_535	Positive	Positive	Field	Cq = 29.5	YES	OBP LSD vaccine	19	Field
LSDV_MKD_187	Positive	Positive	Field	Cq = 31.3	YES	OBP LSD vaccine	17	Field
LSDV_MKD_665	Positive	Positive	Neg	Cq = 35.2	YES	Lumpyvax	16	NA
LSDV_MKD_42	Positive	Positive	Field	Cq = 31	YES	OBP LSD vaccine	15	Field
LSDV_MKD_118	Positive	Positive	Field	Cq = 11	YES	Lumpyvax	12	Field
LSDV_MKD_658	Positive	Positive	Field	Cq = 31.7	YES	OBP LSD vaccine	10	Field
LSDV_MKD_251	Positive	Positive	Neg	Cq = 34.1	YES	OBP LSD vaccine	17	NA
LSDV_MKD_475	Positive	Positive	Field	Cq = 18.5	YES	OBP LSD vaccine	16	Field
LSDV_MKD_493	Positive	Positive	Field	Cq = 26.1	YES	OBP LSD vaccine	15	Field
LSDV_MKD_650	Positive	Positive	Field	Cq = 19.7	YES	OBP LSD vaccine	10	Field
LSDV_MKD_120	Positive	Positive	Field	Cq = 14.6	YES	OBP LSD vaccine	54	Field
LSDV_MKD_393	Positive	Pos (3/4)	Field	Cq = 22.7	YES	OBP LSD vaccine	12	Field
LSDV_MKD_415	Positive	Pos (3/4)	Field	Cq = 14	YES	OBP LSD vaccine	12	NA
LSDV_MKD_429	Positive	Pos (3/4)	Field	Cq = 19.1	YES	OBP LSD vaccine	10	Field
LSDV_MKD_467	Positive	Pos (3/4)	Field	Cq = 25.7	YES	OBP LSD vaccine	11	Field
LSDV_MKD_534	Positive	Pos (3/4)	Field	Cq = 26.6	YES	OBP LSD vaccine	10	Field
LSDV_MKD_553	Positive	Pos (3/4)	MIXED	Cq = 34.6	YES	OBP LSD vaccine	13	Field
LSDV_MKD_563	Positive	Pos (3/4)	Field	Cq = 29.6	YES	OBP LSD vaccine	11	Field
LSDV_MKD_613	Positive	Pos (3/4)	Field	Cq = 28.8	YES	OBP LSD vaccine	11	Field
LSDV_MKD_629	Positive	Pos (3/4)	Field	Cq = 22.8	YES	OBP LSD vaccine	11	Field
LSDV_MKD_621	Positive	Pos (3/4)	Vaccine	Cq = 38.4	YES	OBP LSD vaccine	11	Virulent-Vacc
LSDV_MKD_174	Positive	Positive	Field	Cq = 14.8	YES	OBP LSD vaccine	11	Field
LSDV_MKD_170	Positive	Positive	Field	Cq = 29.35	YES	OBP LSD vaccine	12	Field
LSDV_MKD_176	Positive	Positive	Field	Cq = 25	YES	OBP LSD vaccine	12	Field
LSDV_MKD_211	Positive	Positive	Field	Cq = 38.6	YES	OBP LSD vaccine	14	Field
LSDV_MKD_311	Positive	Positive	Field	Cq = 16.9	YES	OBP LSD vaccine	13	Field
LSDV_MKD_313	Positive	Positive	Field	Cq = 30.6	YES	OBP LSD vaccine	15	Field
LSDV_MKD_316	Positive	Positive	Field	Cq = 39	YES	OBP LSD vaccine	11	NA
LSDV_MKD_357	Positive	Positive	Field	Cq = 22.1	YES	OBP LSD vaccine	14	Field
LSDV_MKD_377	Positive	Positive	Field	Cq = 24	YES	OBP LSD vaccine	10	Field
LSDV_MKD_382	Positive	Positive	Field	Cq = 22.4	YES	Lumpyvax	12	Field
LSDV_MKD_383	Positive	Positive	Field	Cq = 21.9	YES	OBP LSD vaccine	17	Field
LSDV_MKD_388	Positive	Positive	Field	Cq = 14.4	YES	OBP LSD vaccine	12	Field
LSDV_MKD_391	Positive	Positive	Field	Cq = 19.6	YES	OBP LSD vaccine	11	Field
LSDV_MKD_394	Positive	Positive	Field	Cq = 14.4	YES	OBP LSD vaccine	12	NA
LSDV_MKD_587	Negative	Positive	Field	Cq = 11.1	YES	Lumpyvax	13	Field
LSDV_MKD_639	Positive	Positive	Field	Cq = 27.1	YES	OBP LSD vaccine	10	Field
LSDV_MKD_666	Positive	Positive	Field	Cq = 30.7	YES	Lumpyvax	11	Field
LSDV_MKD_672	Positive	Positive	Field	Cq = 40	YES	OBP LSD vaccine	17	NA
LSDV_MKD_689	Positive	Positive	Field	Cq = 29.5	YES	OBP LSD vaccine	11	Field
LSDV_MKD_699	Positive	Positive	Field	Cq = 25.1	YES	OBP LSD vaccine	12	Field
